# Spinal epidural hematoma in a patient on chronic anticoagulation therapy performing self-neck manipulation: a case report

**DOI:** 10.1186/s12998-019-0264-9

**Published:** 2019-09-19

**Authors:** Jesse Cooper, Patrick Battaglia, Todd Reiter

**Affiliations:** 1 Department of Chiropractic Medicine, Baylor Scott and White Health, 300 University Blvd., Building A, Round Rock, TX 78665 USA; 2 Department of Chiropractic, Logan University, 1851 Schoettler Road, Chesterfield, MO 63017 USA; 3 Department of Physical Medicine and Rehabilitation, Novant Health Forsyth Medical Center, 3333 Silas Creek Parkway, Winston-Salem, NC 27103 USA

**Keywords:** Spinal epidural hematoma, Spinal manipulation, Anticoagulants

## Abstract

**Background:**

Spinal epidural hematoma is a rare condition usually secondary to trauma and coagulopathy. To the best of our knowledge, we present the first case of a patient with an iatrogenic hypercoaguable state performing self-neck manipulation, which resulted in a spinal epidural hematoma and subsequent quadriparesis.

**Case presentation:**

A 63-year-old man presented to the emergency department with worsening interscapular pain radiating to his neck 1 day after performing self-manipulation of his cervical spine. He was found to be coagulopathic upon admission, secondary to chronic warfarin therapy for the management of atrial fibrillation. Approximately 48 h after the manipulation, the patient became acutely quadriparetic and hypotensive. Urgent magnetic resonance imaging revealed a multilevel spinal epidural hematoma from the lower cervical to thoracic spine.

**Conclusions:**

Partial C7, complete T1 and T2, and partial T3 bilateral laminectomy was performed for evacuation of the spinal epidural hematoma. Following a 2-week course of acute inpatient rehabilitation, the patient returned to his baseline functional status. This case highlights the risks of self-manipulation of the neck and potentially other activities that significantly stretch or apply torque to the cervical spine. It also presents a clinical scenario in which practitioners of spinal manipulation therapy should be aware of patients undergoing anticoagulation therapy.

## Background

Spinal epidural hematoma (SEH) is an uncommon condition with potential for severe neurological compromise. Differentials for SEH include spontaneous, traumatic, iatrogenic, and coagulopathy related [1]. In rare instances, SEH has followed spinal manipulation therapy (SMT). SMT commonly involves high-velocity, low-amplitude forces applied to articulations of the spinal column. In 2015, Huang et al. [2] presented 12 cases of SEH related to SMT, highlighting the rarity of this clinical phenomenon. Acute (< 24 h) epidural hematoma of the cervical spine following SMT has only been reported 6 times in the English literature [3]. Self-neck manipulation, described as a self-applied force of rapid cervical spine lateral rotation with a “popping” sensation [4], has been linked to cervical artery dissection [5] but just once to SEH [6]. To the best of our knowledge, we present the first case of a patient with an iatrogenic hypercoaguable state performing self-neck manipulation, which resulted in SEH and subsequent quadriparesis. It is critical for health care practitioners to be aware of potential risks when considering SMT as part of a treatment regimen in candidates undergoing anticoagulation therapy.

## Case presentation

A 63-year-old man presented to the emergency department (ED) with unremitting interscapular pain radiating to his neck, which he suspected to be from a myocardial infarction (MI). Onset was 1 day after performing self-manipulation of his cervical spine. He described the maneuver by grabbing his chin and applying a rapid, rotating motion bilaterally. He reported a long history (> 20 years) of performing this maneuver intermittently with perceived benefit. Initial workup in the ED aimed to identify the potential source of pain and consisted of computed tomography angiogram of the chest and abdomen, which excluded dissecting thoracic aneurysm, MI, and pulmonary embolism. Relevant past medical history included atrial fibrillation (AF) managed with long-term warfarin therapy. His international normalized ratio (INR) was 7.84 upon admission to the ED, and he was admitted to the hospital for closer monitoring of any signs or symptoms of neurovascular compromise. The following day, the patient became acutely quadriparetic and hypotensive. His blood pressure decreased to 75/38 mmHg, and his neurological level was determined to be C7 American Spinal Injury Association Impairment Scale D [7].

**Fig. 1:**
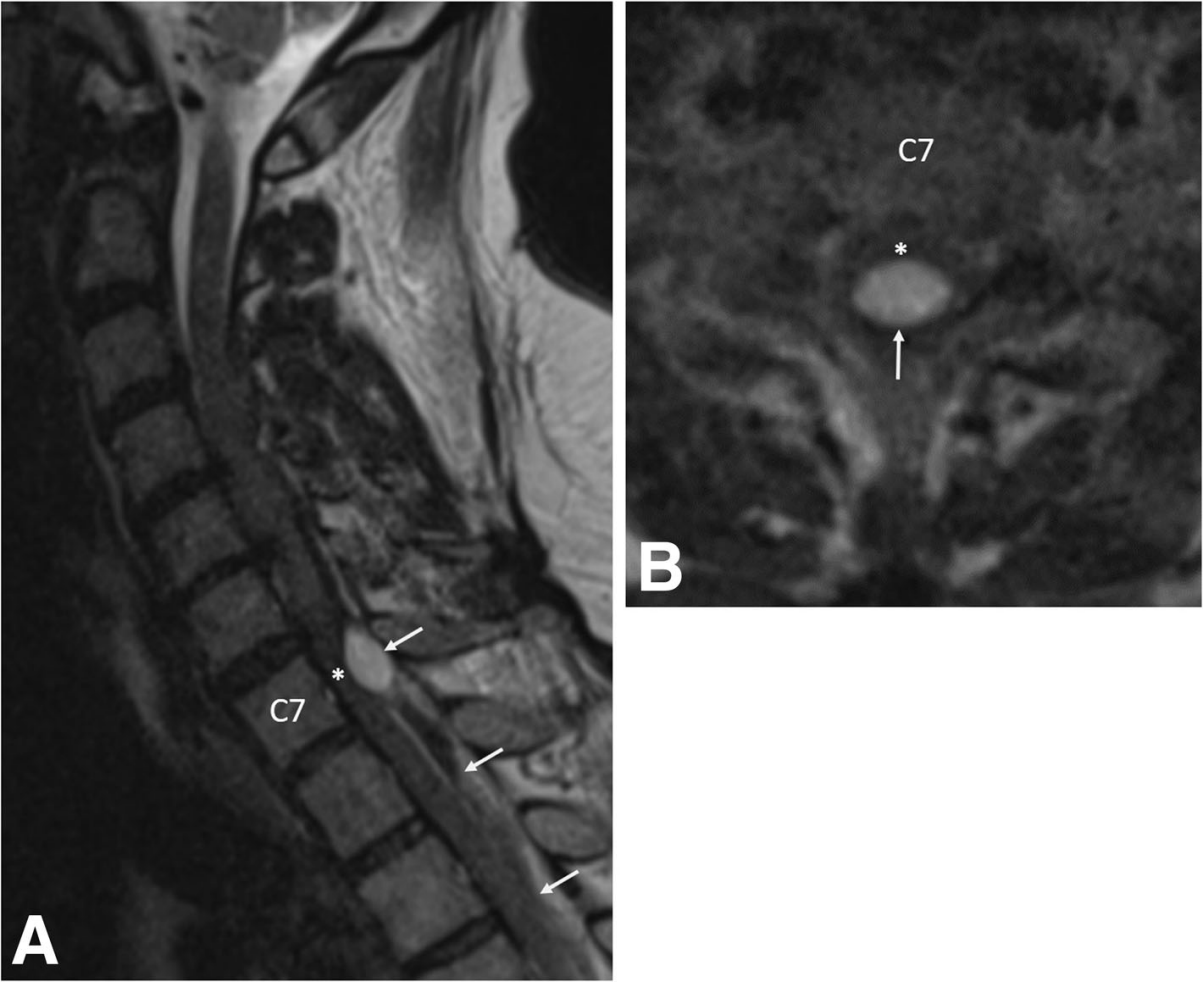
**a** Pre-operative sagittal T2-weighted MRI of the cervical spine demonstrates an epidural hematoma (arrows) with the cranial extent at the C6/7 level. The hematoma is mixed signal intensity, consistent with various stages of bleeding. Severe spinal cord (asterisk) compression is present at the C7 level. **b** Pre-operative axial T2-weighted MRI at the C7 level demonstrates a loculated epidural hematoma (arrow) compressing the spinal cord (asterisk). Due to the urgent nature of the MRI, image quality is degraded

Urgent magnetic resonance imaging (MRI) of the cervical and thoracic spine demonstrated multilevel SEH from the lower cervical to thoracic spine (Fig. 1). With identification of an active bleed and an INR remaining close to 7.0, the patient’s hypercoaguable state was reversed with fresh frozen plasma and factor VII prior to emergency surgery. The patient underwent partial C7, complete T1 and T2, and partial T3 bilateral laminectomy for evacuation of SEH. Placement of an epidural drainage catheter was also performed with the use of C-arm fluoroscopy.

**Fig. 2:**
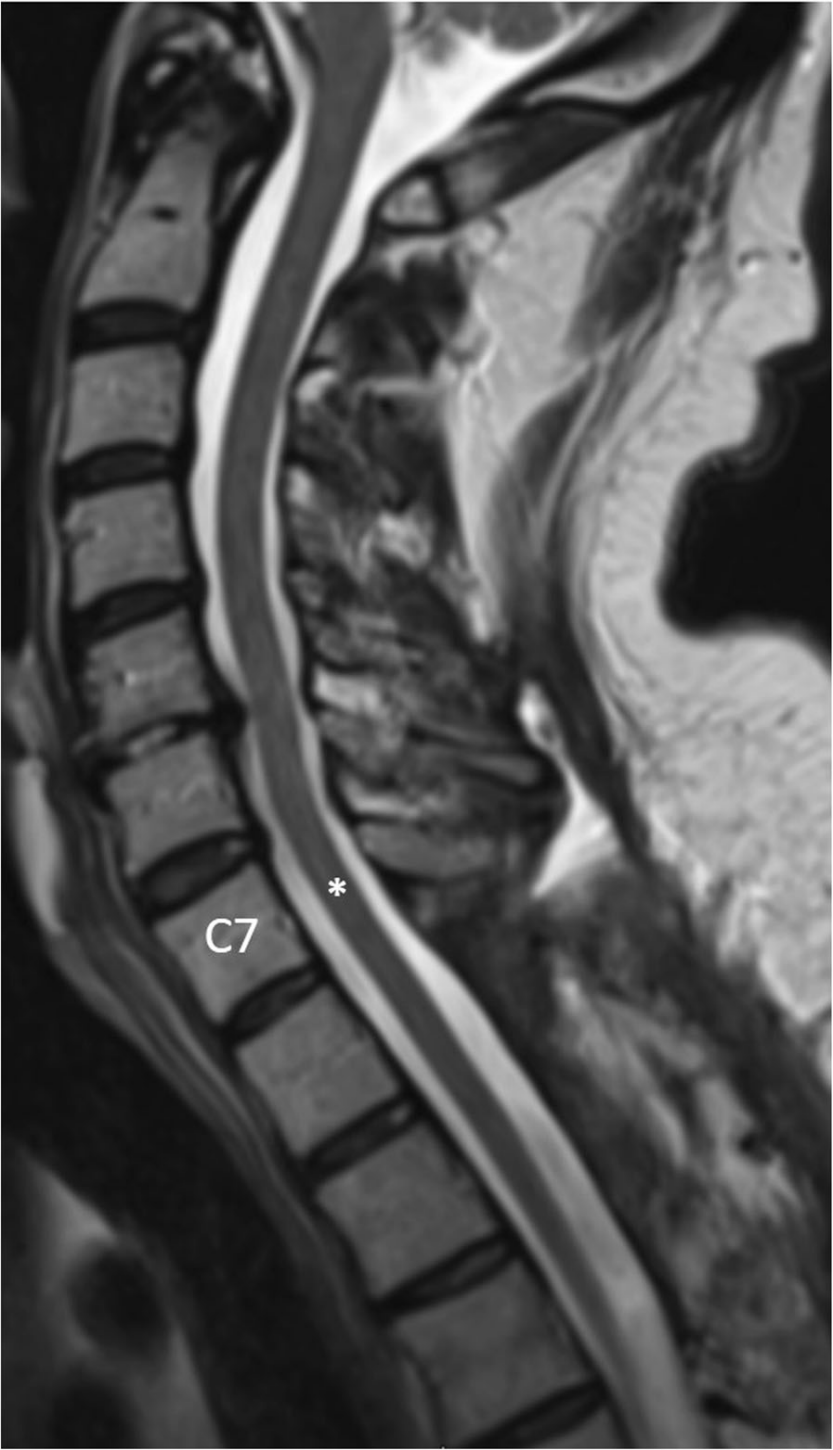
Post-operative sagittal T2-weighted MRI of the cervical spine demonstrates resolution of the epidural hematoma with decompression of the spinal cord (asterisk) at the C7 level. There has been restoration of normal spinal canal volume and there is no evidence of residual cord compression or myelomalacia. Note the absent visualization of the C7, T1, T2, and T3 spinous processes. The patient had partial C7, complete T1 and T2, and partial T3 laminectomy for decompression and hematoma evacuation

Throughout the first 6 days following surgery, the patient recovered some function of the upper and lower extremities while undergoing hospital-based physical therapy. Post-operative MRI showed resolution of the SEH and cord compression without resultant cord damage (Fig. 2). The patient was admitted 7 days post-operatively to an acute inpatient rehabilitation unit with: 1) INR of 1.23; 2) acute kidney injury, which improved with hydration; 3) atrial fibrillation converted to sinus bradycardia on antiarrhythmic medications, not to be anticoagulated; 4) neurogenic bladder secondary to spinal cord injury; 5) diabetes mellitus controlled with medication; 5) and anemia, which improved with iron supplementation. Physical examination revealed 4/5 manual muscle strength testing to the upper and lower extremities. He exhibited moderate dysmetria with finger-to-nose testing and slow initiation of movement. Deep tendon reflexes were 1+/4 to the bilateral lower extremities but 2+/4 to the upper extremities. There were no findings of ankle clonus or pronator drift.

The patient completed a 2-week course of acute interdisciplinary rehabilitation. The epidural drainage catheter remained in place for most of his post-operative stay. His neurogenic bladder with urinary retention resolved. At hospital discharge, the patient’s greatest deficits remained his fine motor skills to his right hand along with residual paresthesia in his lower extremities. He also exhibited mild dyspraxia with finger-to-nose testing on the right and heel-to-shin testing bilaterally. The patient regained full manual muscle strength of all 4 extremities and ultimately returned to his baseline functional status, which included ambulating independently with a cane and independence with activities of daily living.

## Discussion and conclusions

SEH is a rare surgical emergency associated with trauma, coagulopathy, spontaneous development, and iatrogenic procedures, such as spine surgery and spinal punctures [2]. The incidence of SEH is estimated between 0.15 and 1.5 per 100,000 people annually [8]. Even more infrequent, is the incidence of SEH following SMT. This is the first reported case of SEH following self-neck manipulation in a patient on chronic anticoagulation therapy. A report of one case should not suggest causality. It should be noted that SEH symptoms manifested approximately 48 h after the neck manipulation, and the patient had an elevated INR for a sustained period, which may also have been a contributing factor. Adjusted-dose warfarin requires routine monitoring in AF patients to maintain a therapeutic INR target of 2.0 to 3.0, as one of the strongest predictors of major bleeding is INR above 3.0 [9]. The patient in our case did not adhere to routine monitoring standards, rendering him susceptible to a bleed. Although INR monitoring is intimately related to warfarin dosing, practitioners should also understand INR does not apply to patients on non-vitamin K oral anticoagulants, such as dabigatran, rivaroxaban, apixaban, and edoxaban. Nonetheless, this report highlights a potential complication of cervical spinal manipulation and other maneuvers requiring extreme cervical rotation in patients who are over-anticoagulated. This relationship requires further investigation.

In a review of the literature, there is only one case that described self-neck “twisting” temporally associated with development of a cervical epidural hematoma [6]. Interestingly, this self-applied maneuver occurred 2 h after a spinal puncture but in a patient without impaired coagulation. SMT is generally considered a safe procedure but should only be performed by trained health care professionals, following a complete medical history and a thorough physical examination [10]. The incidence of serious complications from SMT, associated with neurological and vascular injury, is estimated between 1 in 400,000 and 1 in 2,000,000 manipulations [11]. The incidence of adverse events from self-neck manipulation is unknown. Risk factors for serious complications of SMT may include failure to identify patients with coagulation disorders. Manipulation of the spine of an over-anticoagulated patient could potentially increase the likelihood of SEH [11].

The mechanism of injury between SMT and SEH may be related to the source of bleeding. Although arterial bleeding has been debated as the origin of hemorrhage in SEH, a considerable body of evidence supports the theory that the venous system is the primary source [12]. The posterior epidural venous plexus is a delicate network of vessels, largely unprotected from trauma or changes in abdominal or thoracic pressure, potentially making it susceptible to rupture. This may explain why a forceful movement, such as self-neck manipulation or SMT, could be capable of injuring vulnerable epidural venous structures. In our case, the patient’s neurovascular deficits clinically manifested as quadriparesis and hypotension approximately 48 h following reported self-neck manipulation. Bleeding from epidural veins without valves could explain this slow progression of spinal cord compression [3].

From a clinical perspective, once the epidural space is breached, the source of hemorrhage does not appear to be a major prognosticator [12]. Regardless of etiology, SEH causes compression of the spinal cord, which can result in irreversible neurological deficits if not treated in a timely manner. The strongest predictor of clinical outcome in SEH patients is directly related to the length of time between ictus and surgical intervention [12]. Although spontaneous resolution has been reported, early diagnosis via MRI and surgical decompression remain the standard of care. The patient in our case likely made a full recovery due to the emergency surgical intervention that occurred promptly after his progressive neurological deficits manifested [3].

The rarity of traumatic SEH makes it difficult to establish cause and effect relationships, though all potential risks of SMT should be communicated and discussed during consenting of patients. Of great clinical importance are the potential reasons why bleeding into the epidural space occurs. Oral anticoagulation use resulting in supratherapeutic INR has become a predominant risk factor [12]. Until the relationship between SEH and manipulation of the spine is better understood, we advise that SMT and exercises or stretches that apply significant torque to the cervical spine should be cautioned in patients with known coagulopathy. Further research is needed to determine whether certain side effects of anticoagulation therapy should be considered contraindications to SMT, necessitating consideration of a more careful clinical course.

## Data Availability

No dataset was generated or analyzed during the current study.

## Abbreviations

AF: Atrial fibrillation
ED: Emergency department
INR: International normalized ratio
MI: Myocardial infarction
MRI: Magnetic resonance imaging
SEH: Spinal epidural hematoma
SMT: Spinal manipulation therapy
